# Dried Powder Formulation of Ksheerapaka for the Management of Primary Dysmenorrhea: A Review

**DOI:** 10.7759/cureus.105535

**Published:** 2026-03-19

**Authors:** Parvathy Unnikrishnan, Prathiksha Rathod, Jyotsana Sanjay Potdar, Pooja Shrivastav

**Affiliations:** 1 Department of Stri Roga and Prasuti Tantra (Gynaecology and Obstetrics), Amrita School of Ayurveda, Amrita Vishwa Vidyapeetham, Amritapuri, IND; 2 Department of Stri Roga and Prasuti Tantra (Gynaecology and Obstetrics), Mahatma Gandhi Ayurved College, Hospital and Research Centre, Datta Meghe Institute of Higher Education and Research (Deemed to be University), Wardha, IND; 3 Department of Gynaecology and Obstetrics, Jawaharlal Nehru Medical College, Datta Meghe Institute of Higher Education and Research (Deemed to be University), Wardha, IND; 4 Department of Shalyatantra (Surgery), Mahatma Gandhi Ayurved College, Hospital and Research Centre, Datta Meghe Institute of Higher Education and Research (Deemed to be University), Wardha, IND

**Keywords:** apana vata imbalance, dried powder formulation, ksheerapaka, primary dysmenorrhea, udavartini yonivyapad

## Abstract

Dysmenorrhea is a prevalent and recurrent gynaecological disorder characterised by menstrual pain arising from inflammatory, neuroendocrine, and functional mechanisms, often inadequately managed by long-term conventional therapies. Ayurveda conceptualises this condition as Udavartini Yonivyapad, attributed to vitiation and disordered movement of Apana Vata, providing a functional framework for holistic management. This review aims to critically examine primary dysmenorrhea from both biomedical and Ayurvedic perspectives and to evaluate the therapeutic relevance of Ksheerapaka, with particular emphasis on the rationale for developing a dried powder formulation to enhance clinical applicability.

A narrative review was conducted using peer-reviewed literature retrieved from PubMed and Scopus, published between 2015 and 2025, focusing on dysmenorrhea, Ksheerapaka, milk-based Ayurvedic formulations, and pharmaceutical dosage form innovations. Evidence from clinical, experimental, and observational studies indicates that Ksheerapaka exhibits analgesic, anti-inflammatory, Vata-pacifying, and tissue-nourishing properties, with consistent benefits reported in gynaecological and pain-related conditions; however, dysmenorrhea-specific clinical evidence remains limited.

The review identifies key pharmaceutical challenges associated with the classical liquid formulation, including limited stability, dosing variability, and reduced patient adherence. Reformulation into a dried powder form is highlighted as a scientifically sound strategy to improve shelf life, standardisation, dosing precision, and usability, while preserving therapeutic intent.

The review concludes that dried powder Ksheerapaka represents a promising integrative approach for primary dysmenorrhea management, but systematic formulation development and targeted clinical evaluation are needed to support evidence-based adoption.

## Introduction and background

Primary dysmenorrhea is a very common gynaecological condition characterised by painful menstruation that can significantly interfere with one's daily activities, academic performance, occupational functioning, and quality of life [1]. In this review, the term dysmenorrhea refers specifically to primary dysmenorrhea, unless otherwise stated, as the present article focuses on menstrual pain occurring in the absence of identifiable pelvic pathology. Dysmenorrhea is broadly classified into primary dysmenorrhea, which occurs in the absence of identifiable pelvic pathology, and secondary dysmenorrhea, which arises due to underlying pelvic conditions, such as endometriosis, adenomyosis, uterine fibroids, or pelvic inflammatory disease. Among these, primary dysmenorrhea is the most prevalent, particularly among adolescents and young women, and is defined by the absence of identifiable pelvic pathology [2].

Epidemiological studies indicate that dysmenorrhea has a high prevalence among menstruating women, particularly adolescents and young adults, with a substantial proportion experiencing recurrent menstrual pain during their reproductive years. Recurrent menstrual pain is associated not only with physical discomfort but also with reduced academic and occupational performance, psychological distress, and altered pain perception [3]. Prolonged cyclical pain has also been shown to result in alterations in central pain modulation and emotional processing, indicating that primary dysmenorrhea represents a multidimensional, chronic health condition rather than an isolated symptom [4].

From a biomedical perspective, the pathophysiology of primary dysmenorrhea is largely attributed to excessive production of endometrial prostaglandins during menstruation [5]. Elevated prostaglandin levels increase uterine contractility, vasoconstriction, and uterine ischemia, which collectively contribute to the characteristic menstrual pain [6]. Additional mechanisms, including vasopressin activity, inflammatory mediators, and oxidative stress, may further exacerbate symptom severity [7]. Conventional treatment approaches, such as nonsteroidal anti-inflammatory drugs and hormonal contraceptives, aim to suppress prostaglandin synthesis or inhibit ovulation [8]. Although widely used, these treatments may be associated with adverse effects, contraindications, incomplete analgesia in some patients, and recurrence of symptoms after discontinuation, which can limit their long-term use [9]. These limitations highlight the need to explore complementary or integrative therapeutic strategies that are effective, safe, and sustainable for long-term management [10].

**Figure 1:**
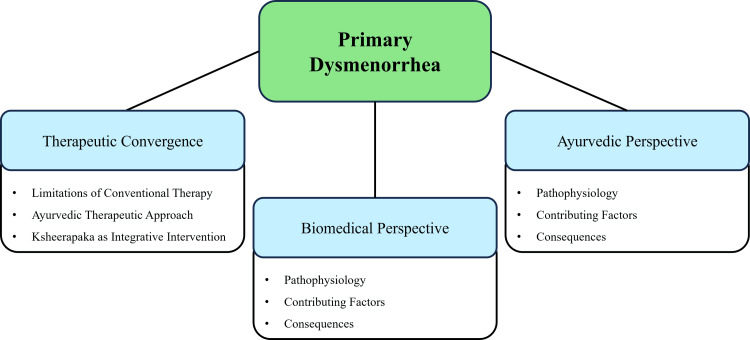
Integrative conceptual framework of primary dysmenorrhea This image was created by the authors using Microsoft PowerPoint (Microsoft Corporation, Redmond, WA, USA).

Within Ayurvedic medicine, primary dysmenorrhea corresponds most closely to Udavartini Yonivyapad, a condition arising from the vitiation and impaired movement of Apana Vata [11]. According to Ayurvedic principles, menstrual pain results from the abnormal upward movement of Vata and may be aggravated by factors such as suppression of natural urges, improper dietary habits, psychological stress, and lifestyle disturbances [12]. In this framework, pain is considered the direct manifestation of aggravated Vata, while irregular or painful menstruation reflects dysfunction of Apana Vata [13]. Management, therefore, focuses on restoring the normal downward movement of Vata, relieving obstruction, and strengthening reproductive tissues, rather than merely suppressing pain [14]. This theoretical framework reflects the chronic and recurrent nature of dysmenorrhea observed in clinical practice [1]. The biomedical and Ayurvedic paradigms intersect in the understanding and management of primary dysmenorrhea, as illustrated in Figure 1.

Ksheerapaka is a classical Ayurvedic dosage form prepared by processing medicinal herbs with milk, usually with the addition of water and controlled heating [15]. It is particularly indicated in conditions dominated by Vata, especially those associated with pain, tissue depletion, and functional weakness [16]. In this preparation, milk serves both as a therapeutic substance and as a pharmaceutical carrier that facilitates the extraction of lipid-soluble and water-soluble constituents, while providing nourishing and Vata-pacifying properties [17]. Consequently, Ksheerapaka formulations are conceptually relevant in the management of primary dysmenorrhea, where menstrual pain is often accompanied by functional impairment and recurrent tissue stress [18]. In Ayurvedic gynaecological practice, such formulations are traditionally employed to alleviate pain, inflammation, and debility, while also promoting systemic nourishment and functional balance [14].

Despite these therapeutic advantages, classical liquid Ksheerapaka presents several practical limitations that restrict its broader clinical use [15]. The formulation has a limited shelf life, requires fresh preparation, and is susceptible to microbial contamination [19]. Issues related to palatability, storage, transportation, and dose variability may also reduce patient compliance and limit formulation standardisation [20]. Variability in preparation methods may further lead to inconsistencies in therapeutic effects, thereby complicating large-scale production and scientific evaluation [19].

In this context, reformulating Ksheerapaka into a dried powder dosage form represents a potential strategy for integrating traditional Ayurvedic principles with modern pharmaceutical approaches [15]. Although direct clinical evidence on dried powder Ksheerapaka for dysmenorrhea remains limited, existing studies on Ksheerapaka in gynaecological and pain-related conditions provide a supportive therapeutic background [11]. Systematic formulation development, standardisation, and targeted clinical evaluation are therefore required to establish its efficacy and safety [19]. Addressing these challenges may enable dried powder Ksheerapaka to emerge as a scientifically validated, patient-adherent, and clinically applicable therapeutic option for the management of primary dysmenorrhea [14].

Objectives of the review

This review aims to critically analyse primary dysmenorrhea from Ayurvedic and biomedical perspectives and to evaluate the therapeutic relevance of Ksheerapaka in its management. It examines the scientific and pharmaceutical rationale for developing a dried powder formulation of Ksheerapaka to overcome limitations of the classical liquid form. The review also finds gaps in evidence and provides future formulation development, standardisation, and future research directions.

## Review

Methodology

A narrative literature review was conducted to identify and synthesise relevant studies related to primary dysmenorrhea, Ayurvedic concepts of Udavartini Yonivyapad, Ksheerapaka formulations, and pharmaceutical development of dosage forms.

Search Strategy

A literature search was performed using electronic databases, including PubMed, Google Scholar, Scopus, and the AYUSH Research Portal. The search strategy combined controlled vocabulary and free-text terms related to dysmenorrhea, Ayurveda, and Ksheerapaka formulations. Boolean combinations such as ("primary dysmenorrhea" OR "menstrual pain") AND ("Ayurveda" OR "Udavartini Yonivyapad"), ("Ksheerapaka" OR "milk decoction") AND ("dysmenorrhea" OR "gynecological disorders"), and ("Ayurvedic formulations" OR "herbal decoction") AND ("dosage form development" OR "spray drying" OR "powder formulation") were used. These combinations were adapted according to the indexing requirements and search functions of each database.

Search Period

The search included studies published between 2000 and 2025, with emphasis on recent literature addressing dysmenorrhea mechanisms, Ayurvedic therapeutic approaches, and pharmaceutical formulation development. The last search was conducted in March 2026.

Eligibility Criteria

Studies were considered eligible if they discussed primary dysmenorrhea or menstrual pain mechanisms, described Ayurvedic concepts of Udavartini Yonivyapad, evaluated Ksheerapaka formulations or related Ayurvedic therapies, or investigated herbal dosage form development, drying technologies, and pharmaceutical standardisation. Articles published in English were included. Publications not related to dysmenorrhea or Ayurvedic therapeutics, reports lacking methodological information, and duplicate records were excluded during screening.

Study Selection and Data Synthesis

Relevant studies were identified through screening of titles and abstracts, followed by full-text assessment. As this article is a narrative review, the objective was conceptual synthesis rather than systematic quantitative aggregation; therefore, database records were not formally enumerated as retrieval counts. Studies were selected based on relevance to dysmenorrhea pathophysiology, Ayurvedic concepts of Udavartini Yonivyapad, therapeutic applications of Ksheerapaka, and pharmaceutical formulation considerations.

Pathophysiology of primary dysmenorrhea: modern and integrative insights

Primary dysmenorrhea is a functional gynaecological disorder characterised by recurrent menstrual pain, with excessive endometrial prostaglandin production representing the most widely established mechanism in the literature [21]. Elevated levels of prostaglandins, particularly prostaglandin F2α and prostaglandin E2, are produced in the endometrium during menstruation and are considered central mediators of the condition [22]. These mediators increase uterine contractility, basal uterine tone, and vasoconstriction, resulting in reduced uterine perfusion and ischemic pain [23]. Elevated prostaglandin concentrations have consistently been associated with increased severity of menstrual pain and remain a key factor in symptom generation in primary dysmenorrhea [22].

Additional mechanisms may also contribute to symptom development. Vasopressin has been implicated in the pathophysiology of dysmenorrhea, as increased circulating levels can further enhance uterine contractions and worsen uterine hypoperfusion [24]. Oxidative stress has likewise been reported to play a role, with increased generation of reactive oxygen species and reduced antioxidant capacity during the menstrual cycle contributing to inflammatory amplification and nociceptor sensitisation [25].

Beyond these peripheral mechanisms, the central nervous system also plays an important role in the persistence and intensity of dysmenorrhea symptoms [26]. Neuroimaging studies have demonstrated altered activation of brain regions involved in pain processing in women experiencing recurrent menstrual pain, suggesting the presence of central sensitisation [26]. Repeated nociceptive input during menstruation may progressively reduce the pain threshold and reinforce pain memory, thereby increasing pain perception over time [27]. Dysmenorrhea is also frequently associated with anxiety, stress, and mood disturbances, which may influence pain perception through interactions with the nervous and autonomic systems [28]. These psychosomatic and neurocognitive factors contribute to variability in symptom severity and the chronic nature of the condition [28].

Overall, primary dysmenorrhea represents a multifactorial disorder involving inflammatory processes, neuroendocrine regulation, central sensitisation, and psychological influences. This complex pathophysiology highlights the limitations of treatment approaches that rely solely on suppression of prostaglandin production [29]. Consequently, comprehensive management strategies that address both peripheral and central mechanisms, while incorporating lifestyle and psychosocial considerations, may provide more effective and sustained symptom control [30].

Ayurvedic pathogenesis of Udavartini Yonivyapad

Primary dysmenorrhea is explained in Ayurveda as Udavartini Yonivyapad, a disorder resulting from the vitiation and abnormal movement of Apana Vata [31]. The principal pathological process involves Vata Prakopa associated with Avarana, which obstructs the normal downward movement of Apana Vata [32]. This disruption in physiological flow leads to painful and difficult expulsion of menstrual blood, with pain typically subsiding once the menstrual flow becomes established [31]. Lifestyle, dietary, and behavioural factors play a central role in the Samprapti (pathogenesis) of Udavartini Yonivyapad [33]. Suppression of natural urges, irregular dietary habits, consumption of dry and cold foods, excessive physical exertion, inadequate rest, and psychological stress are considered key etiological factors that aggravate Vata and promote functional obstruction within the pelvic region [33]. Persistent exposure to these factors sustains Vata vitiation and contributes to the recurrent and cyclical nature of menstrual pain observed in this condition [31].

**Figure 2:**
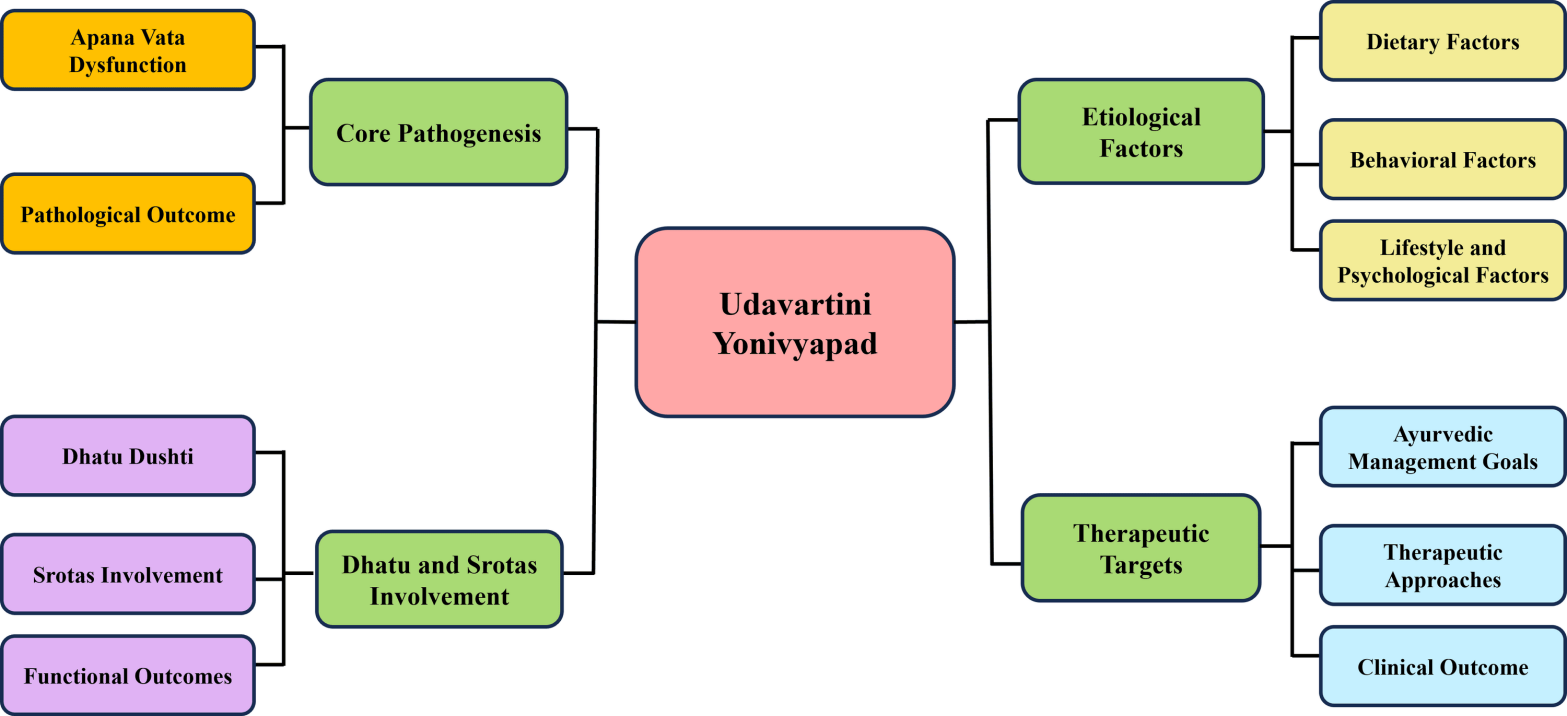
Ayurvedic pathogenesis of Udavartini Yonivyapad This image was created by the authors using Microsoft PowerPoint (Microsoft Corporation, Redmond, WA, USA).

In Ayurvedic theory, aggravated Vata is inherently associated with pain, reflecting the degree and persistence of Dosha imbalance [34]. Involvement of Rasa and Rakta Dhatu is also recognised, as normal menstruation depends on the proper formation, nourishment, and circulation of these tissues [34]. Impairment of these Dhatus may weaken reproductive function and increase susceptibility to pain. Furthermore, dysfunction of Artavavaha and Rasavaha Srotas may lead to altered flow dynamics and functional obstruction rather than structural pathology [31]. Accordingly, the therapeutic objectives in Ayurveda focus on pacifying aggravated Vata, relieving obstruction, restoring the natural downward movement of Apana Vata, and strengthening the affected Dhatus and Srotas [35]. Management strategies, therefore, emphasise Vata-anulomana, Snehana, and Brimhana therapies aimed at correcting functional imbalance and preventing recurrence [35]. This framework reflects the Ayurvedic understanding of primary dysmenorrhea as a disorder of physiological dysregulation with chronic and recurrent manifestations rather than a transient pain symptom [31]. Figure 2 illustrates the etiological factors, core pathogenesis, and therapeutic targets associated with Udavartini Yonivyapad.

Ksheerapaka in Ayurveda: composition, preparation, and indications

Ksheerapaka is a traditional Ayurvedic dosage form prepared by boiling selected medicinal herbs with milk and water until a specified reduction is achieved [15]. This preparation method facilitates the extraction of both aqueous and lipid-soluble constituents, thereby enhancing the therapeutic bioavailability of the formulation [14]. The selection of herbs is guided by the doshic involvement and chronicity of the disease, with priority given to drugs possessing Vata-pacifying and tissue-nourishing properties [16].

Milk serves a dual role in this formulation as both a pharmaceutical medium and a therapeutic agent [17]. According to Ayurvedic principles, its Snigdha, Guru, and Madhura attributes help counteract the dryness, instability, and depletion associated with aggravated Vata [14]. As a lipid-rich medium, milk also facilitates the absorption and tissue penetration of bioactive phytoconstituents while simultaneously supporting systemic nourishment [17]. These characteristics make Ksheerapaka particularly suitable for chronic and recurrent disorders that require prolonged therapeutic intervention [14].

The herbs commonly used in gynaecological Ksheerapaka preparations possess analgesic, anti-inflammatory, antispasmodic, and neuromodulatory properties [16]. Drugs such as Shatavari, Bala, Yashtimadhu, and Dashamoola are frequently included due to their roles in relieving pain, reducing inflammation, modulating neuromuscular activity, and strengthening reproductive tissues [18]. This combined therapeutic approach addresses both symptomatic pain and the underlying functional weakness associated with Vata-dominant disorders [14].

Ksheerapaka is therefore prescribed therapeutically in conditions characterised by Vata predominance, particularly those involving pain, debility, and tissue depletion [16]. In Ayurvedic gynaecological practice, it is widely used in the management of menstrual pain, reproductive weakness, and functional pelvic disorders [11]. Its capacity to provide analgesic effects while simultaneously promoting tissue nourishment and physiological balance supports its therapeutic relevance in the management of primary dysmenorrhea [14].

Evidence on Ksheerapaka in gynaecological and pain disorders

Available clinical and observational evidence suggests that Ksheerapaka-based interventions are widely used in Ayurvedic practice for the management of gynaecological and pain-related disorders [14]. The existing literature primarily consists of case reports, case series, and small-scale clinical studies evaluating the use of oral Ksheerapaka and Ksheerapaka Basti therapies in conditions such as dysmenorrhea, infertility, pregnancy-related ailments, and other Vata-dominant pelvic disorders [11]. These studies typically assess functional outcomes, which align with the Ayurvedic emphasis on restoring physiological balance rather than addressing structural abnormalities [14].

In the context of menstrual disorders, observational clinical reports suggest that Ksheerapaka formulations may contribute to a reduction in pain intensity, improved menstrual flow, and relief of associated symptoms such as pelvic discomfort [11]. Although large-scale clinical trials specifically evaluating oral Ksheerapaka for dysmenorrhea remain limited, available evidence related to Udavartini Yonivyapad and other Vata-mediated gynaecological conditions indicates consistent analgesic and functional benefits associated with this therapeutic approach [31]. Ksheerapaka Basti therapy has also demonstrated beneficial effects in the management of pelvic pain, supporting the therapeutic rationale of milk-based preparations in conditions involving dysfunction of Apana Vata [11].

The therapeutic potential of Ksheerapaka is further supported by its reported applications in other reproductive health conditions. Clinical observations in pregnancy-related care have reported improvements in maternal strength, reduction in abdominal discomfort, and enhancement of overall well-being, reflecting the Brimhana and Balya properties of the formulation [18]. Similarly, studies examining infertility and reproductive debility have described benefits related to tissue nourishment and restoration of reproductive function [16]. Although these findings are indirect in relation to dysmenorrhea, they support the broader applicability of Ksheerapaka in chronic gynaecological conditions characterised by pain and functional impairment [14].

Despite these encouraging observations, the current evidence base has several limitations, including small sample sizes, variability in formulation composition, and heterogeneity in treatment protocols [10]. These factors restrict comparability across studies and limit the strength of clinical inference. Nevertheless, the convergence of positive outcomes across different clinical contexts suggests potential therapeutic relevance of Ksheerapaka in gynaecological disorders [14]. These limitations highlight the need for standardised formulations and well-designed clinical trials to more rigorously evaluate the efficacy of Ksheerapaka in the management of dysmenorrhea [19]. Types of evidence and key clinical outcomes reported for Ksheerapaka-based interventions are summarised in Table 1.

**Table 1:** Clinical evidence on Ksheerapaka in gynaecological and pain-related disorders

Clinical Context	Type of Evidence	Key Outcomes Observed	Reference
Gynaecological and pain-related disorders	Clinical and observational evidence	Widespread use of oral Ksheerapaka and Ksheerapaka Basti therapy in Ayurvedic practice for functional gynaecological conditions	[14]
Dysmenorrhea, infertility, pregnancy-related disorders, Vata-dominant pelvic conditions	Case reports, case series, and small-scale clinical trials	Evaluation of functional outcomes rather than structural correction, consistent with Ayurvedic treatment principles	[11]
Menstrual disorders	Observational reports	Reduction in pain intensity, improvement in menstrual flow, and relief of associated symptoms such as pelvic discomfort	[11]
Udavartini Yonivyapad and Vata-mediated gynaecological disorders	Indirect evidence	Consistent analgesic and functional benefits despite the lack of dysmenorrhea-specific large trials	[31]
Pelvic pain conditions	Therapeutic use of Ksheerapaka Basti	Notable relief of pelvic pain supporting efficacy in Apana Vata dysfunction	[11]
Pregnancy-related applications	Clinical studies	Improved maternal strength and well-being, reduced abdominal discomfort, and enhanced overall well-being, indicating Brimhana and Balya effects.	[18]
Infertility and reproductive debility	Clinical investigations	Improved tissue nourishment and functional restoration	[16]
Overall evidence-based	Methodological assessment	Limitations include small sample sizes, formulation heterogeneity, and variable protocols.	[10]
Cross-condition outcome trends	Comparative outcome analysis	Convergence of positive outcomes across indications suggests therapeutic potential.	[14]
Future research direction	Methodological recommendation	Need for standardised formulations and focused clinical trials in dysmenorrhea.	[19]

Pharmacological rationale of Ksheerapaka ingredients

The pharmacological activity of Ksheerapaka results from the combined effects of its herbal constituents and the functional role of milk as a carrier medium [14]. Herbs commonly used in gynaecological Ksheerapaka formulations possess analgesic, anti-inflammatory, antispasmodic, anxiolytic, and neuromodulatory properties [16]. These pharmacological actions directly correspond to the major pathophysiological mechanisms involved in dysmenorrhea, including uterine hypercontractility, inflammation, ischemia, and altered pain perception [5]. Analgesic and anti-inflammatory effects contribute to the modulation of inflammatory mediators associated with menstrual pain [7], while antispasmodic actions promote relaxation of uterine smooth muscle and reduce ischemic stress [6]. Neuromodulatory effects are particularly relevant because central sensitisation and psychosomatic factors have been reported to influence the severity and persistence of dysmenorrhea symptoms [26]. Through these peripheral and central mechanisms, Ksheerapaka may help address both symptom expression and underlying functional dysregulation associated with dysmenorrhea [14].

Milk functions as a significant synergistic component that enhances the therapeutic potential of the formulation [17]. Its lipid content facilitates the extraction and absorption of fat-soluble phytoconstituents, thereby improving bioavailability and tissue distribution [17]. In Ayurvedic terms, milk possesses Snigdha and Madhura qualities that counteract the dryness and instability associated with aggravated Vata, while its nourishing properties help maintain tissue integrity and neuromuscular stability [14]. This dual pharmaceutical and therapeutic role enhances the suitability of Ksheerapaka for long-term use in chronic conditions [15]. The interaction between herbal constituents and milk, therefore, produces a formulation that is both therapeutically effective and generally well tolerated [14]. Such properties support sustained administration in long-term conditions such as dysmenorrhea, where continuous therapeutic support may be required rather than intermittent treatment [29].

Pharmaceutical challenges associated with conventional liquid Ksheerapaka

Although classical liquid Ksheerapaka is therapeutically appropriate, it presents several pharmaceutical and practical challenges that limit its wider clinical application and standardisation [15]. The high moisture content of the preparation increases its susceptibility to physicochemical instability and microbial contamination, thereby necessitating frequent fresh preparation and careful handling conditions [19]. These characteristics complicate quality assurance, reduce shelf life, and restrict large-scale clinical use [19].

Patient-centred factors also present challenges for the liquid dosage form [20]. The bulkiness of the preparation, storage requirements, and issues related to palatability may reduce patient acceptability, particularly during long-term administration [20]. In addition, the need for repeated preparation or reheating may reduce convenience in outpatient settings and modern healthcare environments, where ease of use and patient adherence are important determinants of treatment compliance [15].

Variability in preparation procedures represents another significant limitation [19]. Differences in raw material quality, processing parameters, and reduction levels during preparation can lead to variations in concentration and dose uniformity [19]. Such variability may affect the reproducibility of therapeutic outcomes and limit the comparability of findings across clinical studies [10]. Irregular dosing further complicates scientific validation and may present challenges in meeting regulatory requirements for standardised medicinal products [19].

These pharmaceutical and operational limitations highlight the need to explore alternative formulation strategies that improve stability, reproducibility, and usability while preserving the therapeutic intent of the traditional preparation [15]. Reformulation through dosage form modification, therefore, provides a rational basis for developing alternative delivery systems, such as dried powder Ksheerapaka, for the management of dysmenorrhea [35].

Dried powder formulations: concepts and pharmaceutical advances

The use of dried powder formulations, especially spray-dried milk powders, has been widely adopted in modern pharmaceutical and nutraceutical development for the improvement of the stability, reproducibility, and usability of liquid-based products [20]. The use of milk powders is a well-established technological precedent that proves that milk-based matrices can be converted into stable, reconstitutable powders while retaining functional and nutritional properties [35]. Moisture reduction achieved by drying has a significant impact on microbial growth and chemical degradation and, therefore, improves shelf life and product reliability [19].

Spray drying is the most widely utilised technique for milk powder production owing to its scalability, reproducibility, and ability to generate uniform particle sizes and a consistent composition [35]. The rapid solvent evaporation in the spray-drying process minimises thermal stress and helps in holding bioactive constituents entrapped in the aqueous milk protein-lipid matrix [35]. Lyophilisation has benefits for thermolabile compounds through low-temperature dehydration by sublimation; however, its higher cost and longer processing time prevent its routine application on a large scale compared with spray drying, especially for milk-based formulations [35]. As an example, advanced technologies in milk powder systems, like microencapsulation, are also used to increase stability, taste masking, and the controlled release of active ingredients (incorporation into protein- or polymer-based matrices) [20].

**Figure 3:**
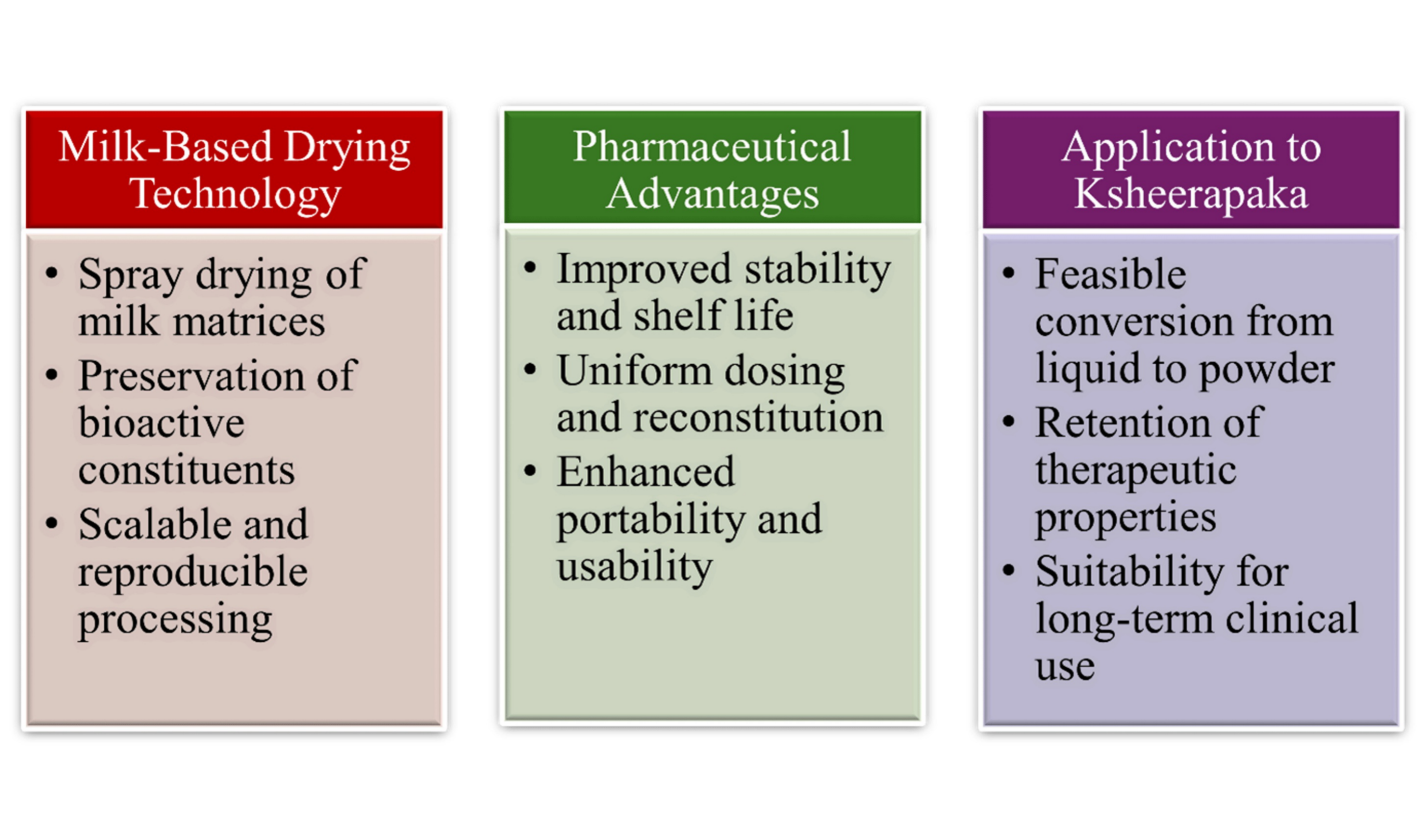
Pharmaceutical rationale and technological basis for dried powder Ksheerapaka using milk-based drying systems This image was created by the authors using Microsoft PowerPoint (Microsoft Corporation, Redmond, WA, USA).

Compared to liquid formulations, milk-based dried powders have superior physicochemical stability, dose uniformity, portability, and predictable reconstitution behaviour [20]. Standardisation of particle size, residual moisture content, and fluid flow characteristics will ensure consistency from batch to batch, which is fundamental when performing scientific analyses and in order to comply with regulations [19]. The ubiquitous pharmaceutical and nutraceutical use of spray-dried milk powders thus affords a good technological basis for the modernisation of traditional milk-based Ayurvedic formulations into modern dosage forms. Within this framework, the conversion of Ksheerapaka into a dried powder is technically feasible, has pharmaceutical justification, and is compatible with long-term clinical use [35]. Figure 3 summarises the benefits, processing methods, and pharmaceutical results of milk-based dried powder formulations relevant to the reformulation of Ksheerapaka.

Scope of dried powder Ksheerapaka in dysmenorrhea management

The conversion of Ksheerapaka into a dried powder formulation provides a practical approach for integrating traditional Ayurvedic therapy with modern pharmaceutical requirements [15]. Classical Ksheerapaka focuses on extracting bioactive constituents within a milk-based matrix to produce Vata-soothing, nourishing, and analgesic effects [14]. This therapeutic matrix can be converted into a stable dried preparation, improving usability and product stability; however, it requires careful standardisation and strict control of critical processing parameters during formulation development [35].

Dried powder Ksheerapaka allows flexible modes of administration [20]. The formulation may be reconstituted with liquid before intake to preserve the traditional method of administration, while alternative dosage forms, such as capsules or granules, may improve patient convenience and treatment adherence [20]. This flexibility is particularly relevant in the management of dysmenorrhea, a condition that often requires sustained and regular therapy over prolonged periods [29]. Improved user-friendliness and consistent dosing may, therefore, enhance long-term patient compliance and therapeutic reliability [20].

From a pharmaceutical perspective, dried powder preparations offer advantages in terms of standardisation and dose precision. Such dosage forms allow more accurate determination of dose and composition, facilitating reproducibility of therapeutic effects and improving comparability across clinical studies [19]. Standardised formulations are essential for reliable evaluation of efficacy, safety, and mechanisms of action in clinical research [19].

Furthermore, dried powder Ksheerapaka may be developed within existing regulatory frameworks governing Ayurvedic or nutraceutical products, thereby supporting wider accessibility while maintaining adherence to classical formulation principles [35]. In this context, dried powder Ksheerapaka represents more than a simple modification of dosage form [15]. It provides a strategy for addressing pharmaceutical limitations associated with traditional liquid preparations while enabling systematic clinical evaluation. Consequently, this formulation approach may support the development of a patient-centred, measurable, and scientifically viable therapeutic intervention for the management of dysmenorrhea [14].

Standardisation, quality control, and regulatory considerations

Standardised measures are essential for developing dried powder Ksheerapaka into a uniform and reliable therapeutic product [19]. This process begins with the authentication and quality assurance of raw herbal materials and milk sources. Subsequently, extraction ratios, drying parameters, and final moisture content must be carefully regulated to ensure formulation consistency [19]. Variations in processing conditions may influence the phytochemical composition and therapeutic reliability of the formulation; therefore, these parameters should be clearly documented within manufacturing protocols and guidelines [19]. Quality control assessment should include physicochemical evaluation, encompassing parameters such as moisture content, particle size distribution, solubility, and reconstitution behaviour [19]. Microbiological testing is particularly important due to the milk-based nature of the formulation, ensuring safety and compliance with permissible microbial limits [19]. In addition, stability studies should be conducted under standardised storage conditions to determine product shelf life and appropriate packaging requirements [19]. Together, these quality control measures establish the basis for reproducibility, safety assurance, and scientific validation of the formulation.

Regulatory considerations also play a critical role in facilitating wider acceptance and utilisation of dried powder Ksheerapaka [35]. Compliance with existing regulatory frameworks governing herbal and traditional medicinal products may support formal recognition and market entry of such formulations [35]. At the same time, preservation of classical Ayurvedic formulation principles remains important to maintain therapeutic integrity [14]. Transparent documentation of manufacturing processes, quality standards, and intended clinical applications can help bridge traditional practices with modern regulatory expectations [35]. A systematic approach to standardisation, quality management, and regulatory compliance, therefore, supports reliable clinical evaluation and promotes integration of dried powder Ksheerapaka into modern healthcare systems [19]. These measures are essential for establishing dried powder Ksheerapaka as a validated and clinically relevant therapeutic option in the management of dysmenorrhea [14]. Table 2 summarises the key quality control measures and regulatory considerations required for formulation validation.

**Table 2:** Standardisation and regulatory considerations for dried powder Ksheerapaka

Aspect	Key Components	Purpose / Outcome	Reference
Standardisation process	Authentication of raw herbs and milk, controlled extraction ratios, drying parameters, and moisture endpoints	Ensures consistency in phytochemical composition and therapeutic reliability	[19]
Manufacturing control	Defined processing protocols and monitored production steps	Minimises batch-to-batch variability and enhances reproducibility	[19]
Physicochemical quality control	Moisture content, particle size distribution, solubility, and reconstitution behaviour	Confirms formulation uniformity, stability, and performance	[19]
Microbiological evaluation	Safety testing specific to milk-based formulations	Ensures microbial safety and compliance with acceptable limits	[19]
Stability assessment	Accelerated and real-time stability studies under standardised storage conditions	Establishes shelf life and appropriate packaging requirements	[19]
Regulatory alignment	Compliance with guidelines for herbal and traditional medicinal products	Facilitates regulatory approval and market integration	[35]
Preservation of Ayurvedic principles	Retention of classical formulation concepts during modernisation	Maintains therapeutic integrity and traditional relevance	[14]
Documentation and transparency	Manufacturing records, quality standards, and intended clinical use	Bridges traditional practice with regulatory expectations	[35]
Clinical and system integration	Standardised, regulated formulation development	Enables reliable clinical evaluation and integration into modern healthcare	[19]
Therapeutic validation	Evidence-based formulation supported by quality and regulatory frameworks	Establishes dried powder Ksheerapaka as a clinically relevant option in dysmenorrhea	[14]

Limitations and future recommendations

Evidence supporting the use of dried powder Ksheerapaka in dysmenorrhea remains limited. Most of the available literature is indirect, drawing primarily from studies on classical liquid Ksheerapaka, or from its application in other gynaecological and pain-related conditions, rather than from dysmenorrhea-specific investigations. Variability in formulation composition, preparation techniques, and treatment protocols also restricts comparability across studies and reduces the reproducibility of reported outcomes. Furthermore, the absence of standardised methods for drying, reconstitution, and dose determination poses challenges for quality assurance and weakens the strength of clinical inference.

Future research should, therefore, prioritise systematic formulation standardisation, including clearly defined processing parameters and validated quality control standards. Well-designed preclinical and clinical investigations are required to establish the efficacy, safety, and consistency of therapeutic outcomes in the management of dysmenorrhea. Comparative studies assessing dried powder Ksheerapaka alongside classical liquid formulations and conventional pharmacological therapies would also help clarify its relative therapeutic advantages. In addition, the integration of appropriate pharmaceutical technologies may enhance bioavailability, reproducibility, and clinical applicability, thereby supporting the evidence-based development of dried powder Ksheerapaka as a viable, long-term therapeutic option.

## Conclusions

This review demonstrates that primary dysmenorrhea is a complex and recurrent gynaecological condition, driven by interconnected inflammatory, neuroendocrine, and functional mechanisms, requiring therapeutic approaches that extend beyond short-term symptomatic relief. Evaluation of Ayurvedic principles alongside contemporary biomedical evidence indicates that Ksheerapaka is a rational formulation for primary dysmenorrhea management due to its Vata-pacifying, analgesic, anti-inflammatory, and tissue-nourishing actions. These effects correspond closely with the Ayurvedic concept of Udavartini Yonivyapad and the biomedical understanding of uterine hyperactivity and pain sensitisation.

Available clinical and observational evidence on Ksheerapaka and related milk-based formulations suggests potential benefits in reducing pain, improving function, and supporting reproductive tissue health; however, condition-specific evidence in primary dysmenorrhea remains limited. Reformulation of classical liquid Ksheerapaka into a dried powder dosage form represents a scientifically justifiable strategy to address limitations related to stability, dosing variability, and patient adherence. Integration of classical formulation principles with contemporary pharmaceutical technologies may enhance standardisation, reproducibility, and clinical applicability. Focused formulation research and well-designed clinical trials are essential to establish dried powder Ksheerapaka as a validated and practical, long-term therapeutic option for primary dysmenorrhea.
